# The Impact of Gender and Age in Obese Patients on Sternal Instability and Deep-Sternal-Wound-Healing Disorders after Median Sternotomy

**DOI:** 10.3390/jcm12134271

**Published:** 2023-06-26

**Authors:** Christian Braun, Filip Schroeter, Magdalena Lydia Laux, Ralf-Uwe Kuehnel, Roya Ostovar, Martin Hartrumpf, Anna-Maria Necaev, Viyan Sido, Johannes Maximilian Albes

**Affiliations:** Department of Cardiovascular Surgery, Heart Center Brandenburg, Faculty of Health Sciences Brandenburg, University Hospital Brandenburg Medical School “Theodor Fontane”, Ladeburger Str. 17, 16321 Bernau bei Berlin, Germany

**Keywords:** surgical-site infection, risk factors, sternotomy, mediastinitis, sternal dehiscence, sternum instability, age, sex, osteomyelitis, sternal wound healing, wound management

## Abstract

Objective: The aim of this study was to investigate the relationship between age and sex in regard to the development of deep sternal wound infections and sternal instability following median sternotomy. Methods: A propensity-score-matching analysis was conducted on 4505 patients who underwent cardiac surgery between 2009 and 2021, all of whom had a BMI of ≥30 kg/m^2^. A total of 1297 matched pairs were determined in the sex group, and 1449 matched pairs we determined in the age group. The distributions of sex, age, diabetes mellitus, delirium, unstable sterna, wire refixation, wire removal, superficial vacuum-assisted wound closure, deep vacuum-assisted wound closure, clamp time, bypass time, logistic EuroSCORE, and BMI were determined. Results: The 30-day in-hospital mortality was found to be similar in the older and younger groups (8.149% vs. 8.35%, *p* = 0.947), and diabetes mellitus was also equally distributed in both groups. However, postoperative delirium occurred significantly more often in the older group (29.81% vs. 17.46%, *p* < 0.001), and there was a significantly higher incidence in men compared with women (16.96% vs. 26.91%, *p* < 0.001). There were no differences found in the incidence of sternum instability, fractured sternum, superficial vacuum-assisted wound closure, and deep vacuum-assisted wound closure between the age and sex groups. Conclusions: In conclusion, this study found that sternal instability and deep-wound-healing problems occur with equal frequency in older and younger patients and in men and women following median sternotomy. However, the likelihood of postoperative delirium is significantly higher in older patients and in men. These findings suggest that a higher level of monitoring and care may be required for these high-risk patient groups to reduce the incidence of postoperative delirium and improve outcomes following median sternotomy.

## 1. Introduction

The most common access route in heart surgery is median sternotomy, which was first performed by Milton in 1897 [1]. After the operation, the breastbone is osteosynthetically stabilized with sternal cerclages. Subsequently, the skin is closed with intracutaneous sutures or skin clips. Deep sternal wound infection is a rare but serious complication after median sternotomy [2]. These deep sternal wound infections must be treated with antibiotics and additional surgical procedures. The most established surgical therapy is debridement and VAC-therapy [3]. Deep sternal wound infections lead to prolonged hospital stays and generate high health costs, placing a burden on the healthcare system and society [2,4,5,6,7]. Ferris et al. calculated the average cost of treating a deep sternal wound infection for the year 2016 to be EUR 62,000. These costs are likely to have increased since then [8]. The morbidity and mortality rates increase significantly, with early mortality and one-year mortality rates rising from 10 to 47% due to deep sternal wound infection. The long-term survival is reduced [2,4,9,10]. In a recently published meta-analysis of 24 studies and 407,829 patients, Perezgrovas-Olaria et al. demonstrated that the mortality rate for deep sternal wound infection is greatly increased [11]. Preoperative risk factors include sex, age, obesity, diabetes mellitus, smoking, chronic obstructive pulmonary disease (COPD), and reduced ejection fraction. Intraoperative risk factors include, in particular, the use of both thoracic arteries (BIMA) and prolonged bypass time. The most important postoperative risk factors are re-exploration of the thorax and blood transfusion [2,6,12,13,14,15,16,17,18,19]. Heilmann et al. described how insulin-dependent diabetes is the most predisposing risk factor for deep sternal wound infection. Heilmann et al. also described how the mechanical stress on the sternum due to COPD is a significant cause of sternal instability but not for superficial-wound-healing disorders [20]. The incidence of deep sternal wound infection varies from 0.6 to 4%. In extreme cases, it can even reach 10% [6,21,22]. Softah et al. reported a rate of 2.5% for superficial-wound-healing disorders and 0.37% for deep sternal wound infections in a study of 3200 patients [23]. For the distinction between a superficial- or deep-sternal-wound-healing disorder, there are essentially two classification systems: first, the classification according to the Centers for Disease Control and Classifications, and second, the Emory classification of sternal wound infection [24,25,26]. In our study, the classification of wound-healing disorders according to the Emory classification was used (see Table 1) [26]. 

Saleh et al. showed that the occurrence of superficial-wound-healing disorders in cardiac surgery is as common as in other surgical disciplines. However, cardiac-surgery patients are more frequently affected by deep sternal wound infections due to the presence of more risk factors [25]. 

Matros et al. found a correlation between deep sternal wound infection and advanced age, while Biancari et al. highlighted the correlation between deep sternal wound infection and female gender [14,27]. 

In our retrospective observational study, we focused on the occurrence of deep sternal wound infection and sternal instability in relation to gender and age.

## 2. Patients and Methods

This study was conducted for quality-control reasons and is completely retrospective in nature. Ethics-committee approval was approved prior to study initiation (E-01-20220705, 18 November 2022). Data collection was fully compliant with the European General Data Protection Regulation (GDPR). After data collection and before statistical analysis, data were anonymized. We conducted a retrospective observational study. Patient consent was waived because of the retrospective study design. We studied 4505 overweight patients, with a body mass index (BMI) of ≥30 kg/m^2^, who had undergone any cardiac surgery with a median sternotomy at our university cardiac institute from 2009 to 2021. Most of them were coronary artery bypass grafting and valve replacements. In the cases of the coronary surgeries, a majority were performed using the left internal mammary artery (LIMA) and the great saphenous vein graft (SVG). The primary objective was to examine age and sex differences regarding the development of deep sternal wound infection and sternal instability after median sternotomy.

### 2.1. Data Collection

In this study, a comprehensive set of baseline data was collected on various demographic and medical factors, including age, sex, body mass index (BMI), presence of diabetes, and the log. EuroSCORE. The female influence on the EuroSCORE was taken into account. These data were used to establish a baseline for comparison with postoperative outcomes. Additionally, specific surgical data, such as aortic clamping time, duration of surgery, and use of the internal mammary artery (IMA), were also recorded. These data are critical in understanding the surgical procedure and its impact on postoperative outcomes.

The occurrence of postoperative delirium, a common complication following cardiac surgery, was assessed using a mini mental test, which is a standard method in clinical practice. Early mortality was defined as death within 30 days post-surgery or patients who never left the hospital. This outcome is a critical measure of the success of the surgical procedure and postoperative care.

Furthermore, data on the occurrence of deep-sternal-wound-healing disorder (DSWI) and surgical-site infections (SIs) were recorded as soon as therapy was required. Information such as when wires needed to be retightened, instances of fractured sternums, and the need for wire removal were also identified. These data provide insight into the healing process of the surgical wound and the interventions required for proper healing. DSWI was treated with negative-pressure wound therapy (NPWT), a well-established treatment for this complication, while SI was treated with retightening of wires, rewiring of the sternum, and secondary closure of the skin wound via back stitches.

Both baseline data and wound-healing data were obtained from the hospital database (SAP^®^, SAP SE, Walldorf, Germany). This ensured the accuracy and completeness of the data. The use of a standardized database also facilitates the analysis and interpretation of the data.

### 2.2. Risk Factor Analysis

In our scientific investigation, we also performed a risk-factor analysis by calculating odds ratios prior to the main study. We distinguished age-related risk factors for the development of deep sternal wound infection and mortality. Moreover, we described gender-related risk factors for mortality and the development of deep sternal wound infection. In both cases, the odds ratios were calculated for each risk factor separately, distinguishing between age groups or sexes where it was adequate.

### 2.3. Statistical Analysis

Statistical analysis was performed using R-4.3.1 for Windows (R Core Team, Vienna, Austria) [28]. To reduce bias for the comparison of age groups (under 80 and over 80 years), as well as males vs. females, we performed propensity-score matchings, using the R-package MatchIt Version 4.5.4, with BMI, logES, and either sex or age as matching parameters [29]. In this technique, a propensity score (PS) is calculated for each patient according to his/her matching parameters before selecting fitting pairs of patients from both study groups that match in their PS. The resulting matched dataset, while reduced in size, aims to minimize the bias usually caused when comparing groups with significantly different baseline characteristics.

Following that, numerical data were tested for normal distribution by using the Shapiro–Wilk test before comparing normal distributed data with Student’s *t*-test and using Mann–Whitney U test otherwise.

Categorical data were compared with Chi^2^ and Fisher’s exact test. In either case, a *p*-value under 0.05 was considered significant.

## 3. Results

### 3.1. Age-Related Risk-Factor Analysis

**Figure 1:**
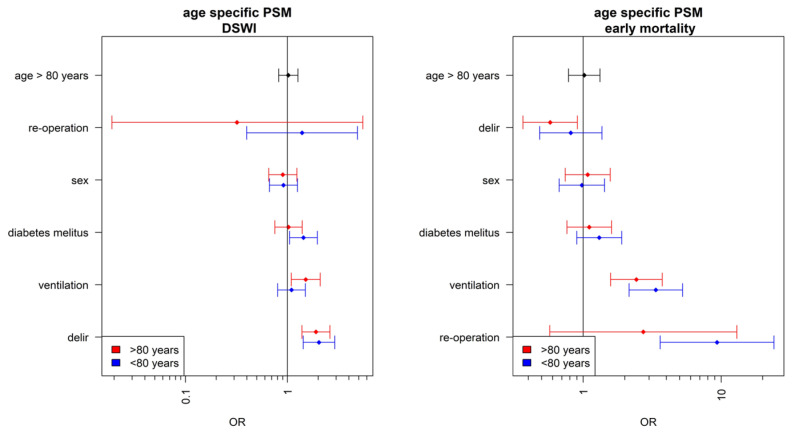
Age-related risk factors. PSM: Propensity Score Matching; DSWI: Deep-Sternal-Wound-Infection.

Diabetes was a significant risk factor for the development of deep sternal wound infection in the under-80 age group. This risk factor was not confirmed for the elderly patients. Patients over 80 years of age had a significantly higher risk for the development of deep sternal wound infection if they received postoperative NIV ventilation (non-invasive ventilation). For patients under 80, NIV ventilation was not a risk factor for developing a deep sternal wound infection. Postoperative delirium was found to be an independent risk factor for the development of deep sternal wound infection across all age groups. Interestingly, it was observed that delirium was not an independent risk factor for mortality in the over-80 age group. Diabetes was a cause of slightly increased mortality in all age groups, but it was not a significant risk factor. However, it was observed that, in all age groups, postoperative NIV ventilation was a significantly independent risk factor for mortality. In patients under 80 years of age, re-operation, i.e., the need for a redo-sternotomy for reasons such as bleeding, was an independent risk factor for increased mortality. This was not a risk factor for those over 80 years old (Figure 1).

### 3.2. Gender-Related Risk-Factor Analysis

**Figure 2:**
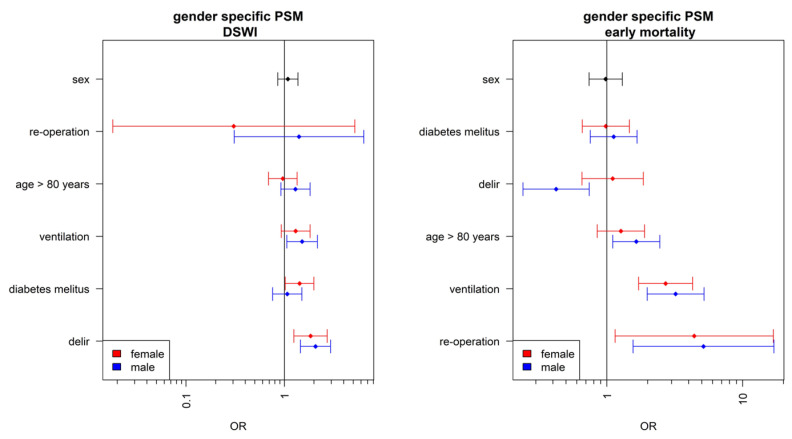
Gender-related risk factors.

Postoperative NIV ventilation was an independent risk factor for the development of deep sternal wound infection in men. In women, the risk for the development of deep sternal wound infection after NIV ventilation was increased, but not significant. Diabetes was only an independent risk factor for the development of deep sternal wound infection in women. Postoperative delirium was found to be an independent risk factor for the development of deep sternal wound infection in both genders. Surprisingly, postoperative delirium was an independent risk factor for lower mortality in men. In women, delirium was not a risk factor for mortality. Older age, over 80 years, was an independent risk factor for mortality in men. This effect was not observed in women. Postoperative NIV ventilation was a significant independent risk factor for mortality in both genders. Re-operation, i.e., the need for repeat sternotomy after primary sternotomy, was an independent risk factor for both genders (Figure 2).

### 3.3. Gender

A total of 4505 patients were included in the study, with 1297 pairs matched for gender. Hospital mortality was found to be equally distributed among females (8.02%, *n* = 104) and males (8.17%, *n* = 106) (*p* = 0.943). Similarly, the incidence of diabetes was found to be equally distributed among females (50.42%, *n* = 654) and males (50.19%, *n* = 651) (*p* = 0.937). However, a significant difference was observed in the incidence of postoperative delirium, with females experiencing a lower incidence (16.96%, *n* = 220) compared to males (26.91%, *n* = 349) (*p* = 0.001). The incidence of unstable sternum was found to be equally distributed among females (3.32%, *n* = 43) and males (4.47%, *n* = 58) (*p* = 0.155). No significant difference was observed in the incidence of sternal wire tightening among females (1.08%, *n* = 14) and males (1.39%, *n* = 18) (*p* = 0.594). A fractured sternum was observed in 1.7% (*n* = 22) of females and 0.85% (*n* = 11) of males, with no significant difference (*p* = 0.08). No significant difference was observed in the incidence of postoperative wire discomfort among females (0.69%, *n* = 9) and males (0.62%, *n* = 8) (*p* = 1). Wire-cerclage removal was required in 5.63% (*n* = 73) of females and 6.4% (*n* = 83) of males, with no significant difference (*p* = 0.457). A total of 3.27% (*n* = 42) of females and 2.7% (*n* = 35) of males required re-sternotomy for various reasons, such as bypass occlusion or bleeding, with no significant difference in distribution of re-sternotomy between the two groups. A superficial VAC-therapy was received by 5.55% (*n* = 72) of females and 5.09% (*n* = 66) of males, with no significant difference (*p* = 0.662). A deep VAC-therapy was received by 2.54% (*n* = 33) of females and 3.01% (*n* = 39) of males, with no significant difference (*p* = 0.55). The aortic clamp time was found to be similar among females (75.28 min ± 45.78 min) and males (72.65 min ± 42.68 min) (*p* = 0.131). However, the bypass time was found to be longer in females (127.2 min ± 61.56 min) compared to males (120.68 min ± 63.99 min) (*p* = 0.008). The log. EuroSCORE was found to be similar among females (11.98 ± 15.75) and males (12.48 ± 15.14) (*p* = 0.411). According to the inclusion criteria, the body mass index (BMI) was above 30 kg/m^2^ in both groups. In the female group, the mean BMI was 34.24 kg/m^2^ ± 4.31 kg/m^2^, and in the male group, it was 34.11 kg/m^2^ ± 4.01 kg/m^2^. This was not found to be statistically significant (*p* = 0.416). The mean age in the female group was 77.15 years ± 9.5 years. In the male group, it was 77.13 years ± 9.72 years. The age was found to be similar in both groups (*p* = 0.948) (Table 2).

### 3.4. Age

The gender distribution was equal among the groups of individuals over 80 years of age and under 80 years of age. Among individuals over 80 years of age, 39.82% (*n* = 577) were female, and among individuals under 80 years of age, 41.82% (*n* = 606) were female. The hospital mortality rate was 8.49% (*n* = 123) among individuals over 80 years of age and 8.25% (*n* = 121) among individuals under 80 years of age, with no significant difference (*p* = 0.947). The prevalence of diabetes mellitus was 52.17% (*n* = 756) among individuals over 80 years of age and 50.1% (*n* = 726) among individuals under 80 years of age, with no significant difference (*p* = 0.281). The incidence of postoperative delirium was 29.81% (*n* = 432) among individuals over 80 years of age and 17.46% (*n* = 253) among individuals under 80 years of age, with a highly significant difference (*p* < 0.001). The incidence of postoperative unstable sternum was 3.86% (*n* = 56) among individuals over 80 years of age and 4.72% (*n* = 67) among individuals under 80 years of age, with no significant difference (*p* = 0.357). The incidence of re-tightening of the wires was 0.97% (*n* = 14) among individuals over 80 years of age and 1.59% (*n* = 23) among individuals under 80 years of age, with no significant difference (*p* = 0.186). The incidence of sternal fracture was 1.31% (*n* = 19) among individuals over 80 years of age and 1.17% (*n* = 17) among individuals under 80 years of age, with no significant difference (*p* = 0.186). The incidence of postoperative wire discomfort was 0.35% (*n* = 5) among individuals over 80 years of age and 0.97% (*n* = 14) among individuals under 80 years of age, with no significant difference (*p* = 0.066). The incidence of postoperative removal of wire cerclage was 6.49% (*n* = 14) among individuals over 80 years of age and 7.04% (*n* = 23) among individuals under 80 years of age, with no significant difference (*p* = 0.605). The incidence of re-sternotomy for any reason, including bleeding or re-operation, was 3.38% (*n* = 49) among individuals over 80 years of age and 3.52% (*n* = 51) among individuals under 80 years of age, with no significant difference (*p* = 0.919). A superficial VAC therapy was received by 5.78% (*n* = 85) of patients in the over-80-years group and 5.52% (*n* = 80) of patients in the under-80-years group. There was no significant difference in this regard (*p* = 0.748). A deep VAC-therapy was received by 3.59% (*n* = 52) of patients in the over-80-years group and 2.69% (*n* = 39) of patients in the under-80-years group. There was no significant difference in this regard (*p* = 0.201). The aortic clamp time in the over-80-years group was 76.52 min ± 49.62 min. The aortic clamp time in the under-80-years group was 72.44 min ± 39.63 min. The aortic clamping time showed a highly significant difference (*p* = 0.014). The bypass time in the over-80-years group was 128.85 min ± 68.4 min. The bypass time in the under-80-years group was 122.92 min ± 59.28 min. The bypass time showed a highly significant difference (*p* = 0.013) (Table 3). 

The heart surgeries took longer in older patients. The log. EuroSCORE in the over-80-years group was 14.29 ± 15.68. In the under-80-years group, it was 12.24 ± 15.59. A highly significant difference was present here (*p* < 0.001). The BMI, according to inclusion criteria, was over 30 kg/m^2^ in both groups. In the over-80-years group, it was, on average, 33.58 kg/m^2^ ± 3.57 kg/m^2^, and in the under-80-years group, it was 33.54 kg/m^2^ ± 3.79 kg/m^2^. This was also not significant (*p* = 0.767). The age in the over-80-years group was 85.17 ± 3.52 years. In the under-80-years group, it was 70.36 years ± 7.73 years. The age was, as expected, significantly different in both groups according to the age-group division (*p* = 0.001).

## 4. Discussion

In many clinical studies, the risk factors that can influence the development of deep sternal wound infections following median sternotomy have been discussed. Biancari et al. found that the female sex is a predictor for the development of deep sternal wound infections [14]. Breyer et al. and Itagaki et al. also demonstrated a significantly higher occurrence of postoperative deep sternal wound infections in women and concluded that the female sex is an independent risk factor for the development of deep sternal wound infections, whereas Krasivskyi et al., in their study, did not see such a difference [30,31,32]. Gatti et al. analyzed 2.872 patients after isolated CABG surgery and showed that the female sex was a strong predictor of DSWI [33]. Copeland et al. gave as the reason for the difference they identified between women and men, and this was that a large female breast exerts inferolateral tension on the incision and, thus, more frequently causes wound-healing disturbances in women. To prevent this, patients were advised to wear a special supportive bra postoperatively [34].

The interaction between CABG and one or both ITAs, along with female sex, were strongly associated with CDC-negative surgical site infection (SSI). Indeed, tension of the breast upon the skin wound may be an additional risk factor for delayed wound healing and wound leakage, leading to SSI [32].

The results of our study were not able to confirm these findings. We found no differences between the sexes regarding the occurrence of unstable sternums and superficial- and deep-wound-healing disturbances. We conducted a single-center match-paired analysis with 1297 pairs, whereas Biancari et al. conducted a prospective multicenter study with a total of 7352 patients [14].

Postoperative delirium can also lead to deep sternal wound infections. In delirium, patients are often disoriented and agitated. The agitation often results in increased shearing forces acting on the freshly operated-on sternum halves. This can lead to deep sternal wound infections and sternum instability. Our results were able to confirm that men are more likely to experience delirium after heart surgery. This could be due to the fact that men have more risk factors that can lead to postoperative delirium than women. For example, men are more prone to excessive alcohol consumption. Perhaps higher testosterone levels might lead to the occurrence of postoperative delirium. To prevent postoperative delirium with the risk of postoperative wound-healing disturbance, patients could be encouraged to reduce the alcohol consumption and take fewer drugs.

The results of the groups of over 80-year-olds and under 80-year-olds were similar to the already known findings from clinical studies.

As expected, the results of our study showed that postoperative delirium occurs more frequently in older patients. Age is an independent risk factor for postoperative delirium [35].

The logistic EuroSCORE was significantly higher in older patients. As older individuals generally have more comorbidities, this is reflected in the EuroSCORE and hospital mortality [36]. However, our study did not show an increased hospital mortality in the group of patients over 80 years of age. It is possible that the group size of approximately 600 patients in both groups was too small to detect a higher hospital mortality in the group of patients over 80 years of age. Both the aortic clamping time and bypass time were significantly higher in the group of patients over 80 years of age. This could be due to the fact that the heart diseases leading to heart surgery in older patients are in more advanced stages than in younger patients. The results of our study are in line with the findings of other groups [37]. With regard to deep sternal wound infection and sternum instability after median sternotomy, no differences in the incidence could be detected. In this regard, our study results differ from those in the research landscape [38]. Our study was not underpowered, with 600 patients in each group. In our heart center, the rate of postoperative wound-healing disorders was the same in old and young patients. Perhaps additional age groups could have been formed to determine the subtle differences in the incidence of wound-healing disorders.

### Limitations

Our study is limited by its retrospective nature. Additionally, it is a single-center study, which may have lower statistical power compared to a multicenter study. The number of cases may be underpowered.

## 5. Conclusions

It has been observed that there is no significant difference in the incidence of sternum instability and deep-wound-healing complications among individuals under 80 years of age and those above 80 years of age, as well as among men and women who have undergone median sternotomy. However, there is a statistically significant increase in the likelihood of postoperative delirium among individuals over 80 years of age and among men. Sternum instability and deep-wound-healing problems occur with equal frequency in under-80- and over-80-year-olds and in men and women undergoing median sternotomy. 

## Figures and Tables

**Table 1:** Emory classification of sternal wound infection.

Type	Depth	Description
1a	Superficial	Skin and subcutaneous-tissue dehiscence
1b	Superficial	Exposure of sutured deep fascia
2a	Deep	Exposed bone, stable wired sternotomy
2b	Deep	Exposed bone, unstable wired sternotomy
3a	Deep	Exposed necrotic or fractured bone, unstable, heart exposed
3b	Deep	Type 2 or 3 with septicemia

**Table 2:** Gender.

	Female	Male	*p*-Value
Sex	100% (1297)	0% (0)	<0.001
In-house death	8.02% (104)	8.17% (106)	0.943
Diabetes mellitus	50.42% (654)	50.19% (651)	0.937
Delirium	16.96% (220)	26.91% (349)	<0.001
Sternum instability	3.32% (43)	4.47% (58)	0.155
Re-fixated wires	1.08% (14)	1.39% (18)	0.594
Fractured sternum	1.7% (22)	0.85% (11)	0.08
Wire discomfort	0.69% (9)	0.62% (8)	1
Wire removal	5.63% (73)	6.4% (83)	0.457
Re-sternotomy	3.24% (42)	2.7% (35)	0.488
Superficial vacuum therapy	5.55% (72)	5.09% (66)	0.662
Deep vacuum therapy	2.54% (33)	3.01% (39)	0.55
Aortic clamping time	75.28 ± 45.78	72.65 ± 42.68	0.131
Bypass time	127.2 ± 61.56	120.68 ± 63.99	0.008
log. EuroSCORE	11.98 ± 15.75	12.48 ± 15.14	0.411
BMI	34.24 ± 4.31	34.11 ± 4.01	0.416
Age	77.15 ± 9.5	77.13 ± 9.72	0.948

BMI: Body Mass Index.

**Table 3:** Age.

	Over 80 Years	Under 80 Years	*p*-Value
Sex	39.82% (577)	41.82% (606)	0.29
In-house death	8.49% (123)	8.35% (121)	0.947
Diabetes mellitus	52.17% (756)	50.1% (726)	0.281
Delirium	29.81% (432)	17.46% (253)	<0.001
Sternum instability	3.86% (56)	4.62% (67)	0.357
Re-fixated wires	0.9% (14)	1.59% (23)	0.186
Fractured Sternum	1.31% (19)	1.17% (17)	0.867
Wire discomfort	0.35% (5)	0.97% (14)	0.066
Wire removal	6.49% (94)	7.04% (102)	0.605
Re-Sternotomy	3.38% (49)	3.52% (51)	0.919
Superficial vacuum therapy	5.87% (85)	5.52% (80)	0.748
Deep vacuum therapy	3.59% (52)	2.69% (39)	0.201
Aortic clamping time	76.52 ± 49.62	72.44 ± 39.63	0.014
Bypass time	128.85 ± 68.4	122.92 ± 59.28	0.013
log. EuroSCORE	14.29 ± 15.68	12.24 ± 15.59	<0.001
BMI	33.58 ± 3.57	33.54 ± 3.79	0.767
Age	85.17 ± 3.52	70.36 ± 7.73	<0.001

## Data Availability

Data will not be published for privacy reasons and will be saved at the hospital.
